# P62/SQSTM1 beyond Autophagy: Physiological Role and Therapeutic Applications in Laboratory and Domestic Animals

**DOI:** 10.3390/life12040539

**Published:** 2022-04-06

**Authors:** Maria Giovanna Sabbieti, Andrea Marchegiani, Albert A. Sufianov, Vladimir L. Gabai, Alexander Shneider, Dimitrios Agas

**Affiliations:** 1 School of Biosciences and Veterinary Medicine, University of Camerino, 62032 Camerino, Italy; giovanna.sabbieti@unicam.it (M.G.S.); andrea.marchegiani@unicam.it (A.M.); 2 Federal Center of Neurosurgery, 625032 Tyumen, Russia; sufianov@gmail.com; 3 Sechenov First Moscow State Medical University, 119991 Moscow, Russia; 4 CureLab Oncology Inc., Dedham, MA 02026, USA; vgabai@curelab.com (V.L.G.); ashneider@curelab.com (A.S.); 5 Department of Molecular Biology, Ariel University, Ariel 40700, Israel; 6 Institute of Biomedical Systems and Biotechnology, Peter the Great St. Petersburg Polytechnic University, 195251 St. Petersburg, Russia

**Keywords:** p62/SQSTM1, inflammation, aging, inflammatory diseases, degenerative diseases

## Abstract

Inflammation is the preceding condition for the development of mild and severe pathological conditions, including various forms of osteopenia, cancer, metabolic syndromes, neurological disorders, atherosclerosis, cardiovascular, lung diseases, etc., in human and animals. The inflammatory status is induced by multifarious intracellular signaling cascades, where cytokines, chemokines, arachidonic acid metabolites, adhesion molecules, immune cells and other components foster a “slow burn” at a local or systemic level. Assuming that countering inflammation limits the development of inflammation-based diseases, a series of new side-effects-free therapies was assessed in experimental and domestic animals. Within the targets of the drug candidates for quenching inflammation, an archetypal autophagic gear, the p62/sqstm1 protein, has currently earned attention from researchers. Intracellular p62 has been recently coined as a multi-task tool associated with autophagy, bone remodeling, bone marrow integrity, cancer progression, and the maintenance of systemic homeostasis. Accordingly, p62 can act as an effective suppressor of inflamm-aging, reducing oxidative stress and proinflammatory signals. Such an operational schedule renders this protein an effective watchdog for degenerative diseases and cancer development in laboratory and pet animals. This review summarizes the current findings concerning p62 activities as a molecular hub for cell and tissues metabolism and in a variety of inflammatory diseases and other pathological conditions. It also specifically addresses the applications of exogenous p62 (DNA plasmid) as an anti-inflammatory and homeostatic regulator in the treatment of osteoporosis, metabolic syndrome, age-related macular degeneration and cancer in animals, and the possible application of p62 plasmid in other inflammation-associated diseases.

## 1. Introduction

Inflammation is the main cause of the most common debilitating and degenerative diseases in humans and animals. Inflammatory disorders include a broad spectrum of acute or chronic, local or systemic disturbances, which drastically impact quality of life. Inflammatory responses remain highly heterogeneous but well-aligned within the following mechanistic scheme: exogenous or endogenous inflammatory inducers activate specific sensors, which mediate the release of biofactors at the target tissue(s). The homeostatic disruptions caused by the “burn signals” can modify main metabolic activities, including core body temperature, sleep patterns, nociceptive thresholds, vascular and airway smooth muscle tone and stress perception. [1,2] Persistent inflammatory signals act as infection and chronic disease incubators, inducing multiple pathological scenarios. Certainly, inflammation has been portrayed as the common soil of the multifactorial maladies [2]. To mention a few, osteoarthritis, osteomyelitis, age-related macular degeneration, atherosclerosis, metabolic syndromes and various forms of cancer fall into the category of inflammatory-based diseases [3]. Currently, treatments approved to alleviate inflammatory processes are steroidal and non-steroidal anti-inflammatory drugs (NSAIDs), whose constant administration can lead to deleterious side effects. Thus, the development of more-efficient and safer anti-inflammatory agents appears essential in suppressing inflammatory cascades. These agents have to deal with complex local lesion formations and prevent the release of pro-inflammatory molecules, e.g., cytokines, chemokines, arachidonic acid metabolites, vasoactive mediators and hydrolytic enzymes [3].

In the last decade, the P62/SQSTM1 protein was considered as a promising therapeutic candidate within an autophagic scenario, or as a molecular hub for multiple signaling pathways. The first functional task assigned to this protein was related to its crucial role in autophagic apparatus. p62 assists selective macroautophagy and acts as a molecular sentinel in the autophagosome membrane for the recognition, sequestration and degradation of intracellular wastes [4,5]. The intramural duties of p62 were not limited to the well-defined cell preservation process, “self-cannibalism”, but extended to various metabolic signaling cascades. For instance, p62 was involved in the NF-κB, mTOR and Wnt pathways, steering the fine line between autophagy and cancer [6], and activates MAP kinases, TRAF6, and Twist1 for cell maintenance, proliferation and migration [7,8].

Physiological p62 levels guarantee the cell steady-state operational agenda, while impaired p62 protein expression interrupts a whole range of metabolic tasks at local and systemic scales. Therefore, p62 overexpression was associated with tumorigenesis, including breast cancer [9], lung cancer [10], osteosarcoma [11] and other cancers. Instead, p62 absence impairs bone and bone marrow dynamics by dysregulating the hematopoietic and mesenchymal stem cell pool, adipocytes congregation and pro-inflammatory molecules release within bone marrow reservoirs [8,12,13]. Undoubtedly, p62 plays a critical role on myeloid lineage homing, maintenance and differentiation fate, as well in bone progenitors’ expansion and maturation [8,12].

In recent years, the most unexpected discovery concerning p62 tasks on cells homeostasis might be the ability of this protein to recruit a wide range of anti-inflammatory cytokine and chemokines for quenching the “slow burn” and (at least partially) to reverse the chronic inflammation [8,13,14,15]. Taking into consideration the various forms of inflammation-based diseases in small animals affecting muscle-skeletal apparatus such as feline poliomyelitis and discospondylitis [16], myopathies, osteochondrosis dissecans and osteopathies in horses and dogs [17,18], but also cutaneous diseases (i.e., canine sterile granulomatous dermatitis) and intestinal T cell lymphoma [17], the development of new and efficient anti-inflammatory drugs becomes a critical knot to untie for field researchers. In context, the p62 functional dimension can lay the groundwork for building an effective platform against inflammation by taking advantage of the multitask intracellular p62 features to improve specific homeostatic tasks in several tissues. Henceforward, we provide an up-to-date analysis of the “handy-protein” p62 within a steady state or in a pathological scenario.

## 2. P62 as a Multitask Protein for Tissue Homeostasis

### 2.1. P62 in Rodent’s Bone and Bone Marrow Pathophysiology

Bone quality is defined as series of architectural and structural parameters, such as mineral composition, cortical porosity and hydroxyapatite crystals geometry, which contribute to bone strength and consistency. The disruption of these parameters due to genetic abnormalities, mineral/hormonal imbalances, inflammation, aging and environmental/lifestyle-related factors leads to osteopathies characterized by an elevated fracture incidence [19,20]. For instance, dogs can foster developmental bone diseases, such as osteomyelitis and nutritional osteopathies, and mammals generally share a fairly similar clinical scenario in this regard (albeit the specific peculiarities of different species and strain must be considered) [21].

Well-defined experimental protocols and treatments were recently tested on animals, targeting metabolic bone diseases and, largely, various form of osteopenia. The most efficient models for studying skeletal disorders encompass mouse, rats, dogs and female sheep (ewes). The ovariectomized (OVX) rat has been portrayed as an excellent preclinical animal model, which properly mimics the osteopenic scenario of estrogen deficiency. Moreover, the immobilized rat model consists of an alternative bone-loss model. The OVX mouse, the glucocorticoid-treated mouse and the senescence-accelerated (SAM/P6) mouse are additional models of severe osteoporosis [22], which are extensively explored by field researchers.

P62 protein plays a conspicuous role in bone metabolic features. Early findings initially correlated p62 protein with the differentiation/commitment of myeloid progenitors to osteoclasts via Nfatc1/NF-κB activation [23]. Afterwards, several studies were aligned with the stance that impaired p62 function leads to enhanced osteoclastogenesis and subsequent bone loss [24,25,26]. An elegant interpretation of these conflicting findings has lately been proposed by Zach and colleagues [27], who attributed p62 to a key role in modulating monocyte differentiation. Specifically, p62 may sustain a more naïve status of monocytes, while p62 absence prompts monocytes maturation toward osteoclasts in murine models [27].

Likewise, young chimeric mice knockout for p62 displays a reduced femoral trabecular volume and number characterized by osteopenia. The p62 molecular and mechanistic mode of action on these animals was both straightforward or/and indirect. Specifically, p62 acts directly by regulating the nuclear factor κB (NF-κB) signaling cascade in osteoblasts, which is indispensable for pre-osteoblasts commitment to mature bone cells. P62 loss in 6- to 8-week-old mice increases IKK/NF-kB activity, with a consequent interruption of the bone-forming cells’ differentiation schedule and the abolishment of osteoblasts sensitivity to C-C motif chemokine ligand 4 (CCL4) and C-X-C motif chemokine 12 (CXCL12) chemotaxis gradients. Nonetheless, a significant reduction in bone progenitor cell differentiation was observed in in p62^−/−^ mice, and thus enfeebled osteogenesis [12].

Additional reports indicated that p62 absence causes weaker PTH osteoanabolic effects [15]. Moreover, in mesenchymal stem cells (MSCs) from p62^−/−^ mice, the survival/bone-building PTH/TGF-β/BMP-related signaling was partially abrogated, and a shift of the MSCs differentiation fate toward adipogenesis was revealed. Thus, p62 absence might foster an inflammatory setting within bone marrow due to fat cells accumulation and pro-inflammatory cytokines/chemokines release [8]. It is also worth bearing in mind the importance of the interconnectedness and interdependence of bone marrow inhabitants, which form well-defined operative compartments within marrow confines. Therefore, the compromised stromal phenotype observed in p62^−/−^ mice due to enhanced adipogenesis, further support the above findings.

Doubtless, p62 partakes as an indispensable gear in homeostatic bone machinery. Specifically, both the contribution of p62 as operating hub for MSCs’ osteo-commitment, and its accomplishment in maintaining the spatial and functional niche cohesion of bone marrow [13,28], fully support the above posture.

**Figure 1:**
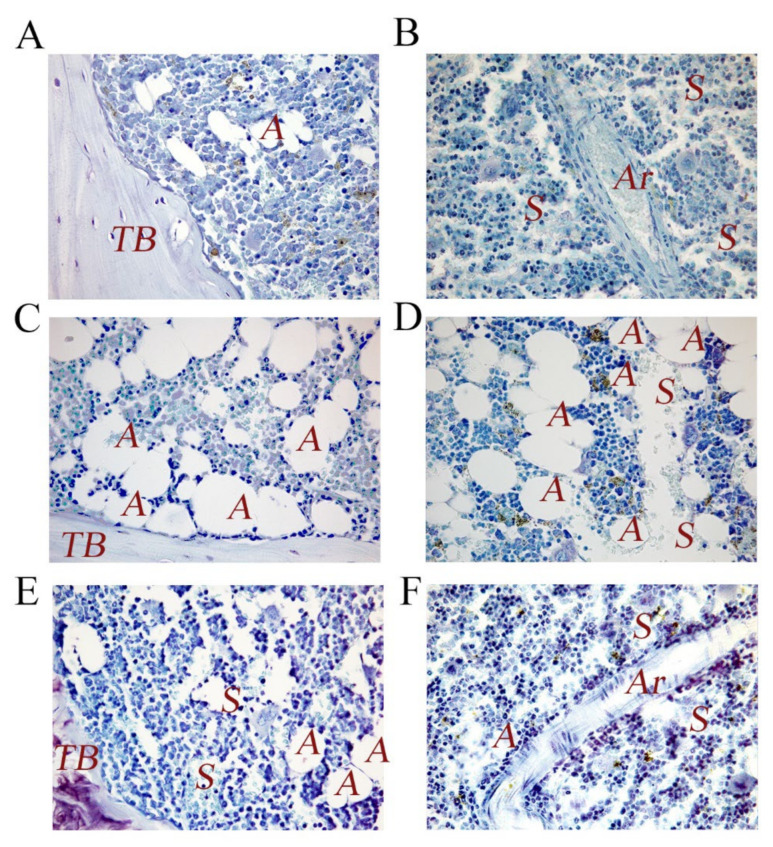
Representative images of femur bone marrow (sub-metaphyseal area) from WT and p62^−/−^ 6-month-old mice stained with toluidine bleu. (**A**) Bone marrow from WT mice. Note the different hematopoietic and stromal cell populations partakes in endosteal niche and the anatomical and structural cohesion and expansion of these bone marrow inhabitants. (**B**) Bone marrow from WT mice. Bone marrow elements surround arterioles and sinusoids to form the perivascular niche. Note the levels of hematopoietic and mesenchymal sub-populations, which reside next to the vessels. (**C**) Bone marrow from mice p62^−/−^. Note adipocytes infiltration and endosteal niche or (**D**) perivascular niche disruption. (**E**,**F**) Bone marrow from p62^−/−^ mice treated with p62DNA plasmid as previously described [11]. Note the decreased fat cells number and the tendency to stabilize a steady state bone marrow phenotype. TB: trabecular bone; A: adipocyte; Ar: arteriole; S: sinusoids. Magnification, ×40.

During our studies of osteoporosis in rodents, we made an unexpected observation that exogenous, rather than endogenous, p62 can also affect bone development. To express exogenous p62, we used the DNA plasmid approach. Originally, DNA plasmids were employed as vaccines to generate immune response to specific protein antigens. Later, they also began to be used as a gene therapy, e.g., to replace defective proteins. We unexpectedly found that p62 plasmid administration demonstrated properties unrelated to immune response and gene therapy. As we observed, exogenous p62 DNA administration could “instruct” MSCs to release growth and differentiation factors, as well as anti-inflammatory cytokines, that together exerted a bone-forming action. Accordingly, p62 DNA-plasmid injection to OVX mice reversed the osteopenic/inflammatory milieu and restored bone micro-architecture via new bone deposition. In particular, p62 DNA prompted a twofold action by inducing the transcription of main pre-osteoblast differentiation markers and by quenching inflammation via anti-inflammatory cytokines/chemokines production. In summary, p62 DNA plasmid has been coined as an effective therapeutic platform to restore the physiological remodeling of age-related osteopenia in mice (Figure 1) [14].

The overall promising p62-related osteo-building breakthroughs obtained in rodents may pave the way for clinical testing in dogs and horses with chronic bone inflammatory diseases. For instance, osteoarthritis is the most common inflammatory disease in dogs with the highest incidence in large breeds and older dogs [29]. To our knowledge, traditional treatments against canine osteoarthritis comprise non-steroidal anti-inflammatory drugs (NSAID) [29,30] and corticosteroids [31]. They are both often accompanied by significant side effects such as vomiting, abdominal pain and withdrawal syndromes [30,31]. Alternative medications such as oral doxycycline [32] or insulin growth factor-I (IGF-I) [33] have been proposed for the management of canine and feline osteoarthritis, as well as nutritional supplementation [34] without encountering the hoped-for success. For these reasons, the development of new therapeutic protocols is extremely important. A recently proposed method to treat inflammatory osteoarthritis in dogs is based on the intra-articular injection of autologous MSCs [35] which are shown to be efficient in decreasing pain and improving the life of dogs. However, this therapeutic approach shows a certain degree of invasiveness. Therefore, considering the anti-inflammatory function of p62 DNA, this could be an alternative, viable and less-invasive therapeutic strategy.

### 2.2. P62 in Metabolic Dysfunctions in Rodents

Obesity is depicted as a chronic and systemic inflammatory disorder, which provokes behavioral and hormonal alterations. Obese animals present a glucose imbalance and frequently develop type 2 diabetes in addition to protein and lipid metabolism imbalance, which can lead to cardiovascular failure [36,37].

A high-calorie diet (HCD) in animals incites a cascade of interactional and metabolic events and might prompt the imbalance of central serotonin levels, fat accumulation and systemic inflammation [38,39]. Precisely, excessive feeding leads to brain dopamine and serotonin dysregulation, which in turn alters the feeling of satiety. Therefore, the hyperphagic status observed after HCD is part of a positive feedback loop between the serotonergic system and the pro-inflammatory cytokines produced by fat cells [38,40]. Of interest, serotonin suppression causes neurological dysfunctions such as anxiety and depression [41]. In specific cases [42], depression prompts food consumption with concurrent adipose tissue accumulation and the systemic release of pro-inflammatory molecules [43], in this way, generating a vicious cycle established among brain and periphery.

Recent findings focused on p62 significance to alleviate metabolic disorders in rats. Namely, when rats were nourished with HCD, it provoked the disruption of glucose metabolism and the generation of pre-diabetic littermates. The treatment with p62 plasmid intramuscularly injected once a week for 6 weeks in this rat model revealed promising outcomes: it (i) decreased and nearly normalized pro-inflammatory cytokines/chemokines, such as IFN-γ, IL-1β and IL-12; (ii) enhanced the secretion of anti-inflammatory cytokines, such as IL-4, IL-10 and TGF-β; (iii) decreased concentrations of glucose, insulin and HbA1c with the concurrent partial reversion of glucose intolerance; and (iv) partially reestablished brain serotonin and tryptophan levels with, presumably, a behavioral status restitution [44].

Overall, p62 plasmid administration was an efficient therapeutic platform able to alleviate the pre-diabetic state of HCD rats and improve the deteriorated/inflamed animal metabolic setting induced by obesity.

In this context, the loss-of-function studies of endogenous p62 have further elucidated and supported the peculiar role of this protein hub in rodents’ metabolic dysfunctions. Employing p62 knock out mice, Rodriguez and collaborators [45] evidenced that these mice were overweight and characterized by distressed glucose metabolism. Other findings highlight that macrophage-specific p62 loss causes a noticeable amassing of damaged mitochondria and an excessive IL-1β-dependent inflammation in mice. Certainly, p62 ablation in macrophage enhances pro-inflammatory IL-1β production, stimulating inflammasome activation. These data suggest that p62 may act as a sentinel for damaged mitochondria and endorses their autophagic clearance [46]. Therefore, p62 might be able to counter sterile inflammation and fulminant hepatitis in mice, and thus this protein has been coined as the “missing link” that boosts NF-κB response to suppress NLRP3-depended inflammasome activation [46]. Of note, the anti-inflammatory function of endogenous p62 may be unrelated to its exogenous functions, and this requires further research.

### 2.3. P62 Protective Effects in Neurodegenerative Ocular Diseases

Inherited retinal degenerations (RDs) embrace a set of diseases that cause blindness in dogs and in humans [47]. Among them, the most common is the age-related macular degeneration (AMD), which is characterized by distinct clinical signs and incites cecity in elderly animals and humans [47,48]. The resurgence factors of AMD implicate metabolic, genetic, environmental and functional parameters, and the progression of the disease is age-related [49]. Consequently, the weakened immune and autophagic responses alongside the oxidative stress and “inflamm-aging” processes encountered in older individuals, can accelerate AMD pathogenesis [50]. It has been found that endogenous p62 plays a significant role in retinal pigment epithelium (RPE) metabolism, since this protein supports the functional aspects of the tissue autophagic apparatus and prompts antioxidant activity to contrast with acute oxidative stress [51]. Pre-clinical trials also addressed the effects of p62 plasmid in a model of senescence-accelerating OXYS rats, an animal model that spontaneously develops AMD-like retinopathy with clinical signs more evident at 12 months or later [52]. The preventive administration of p62 in the young (3 months) OXYS rats demonstrated an efficacy in blocking the advancement of the disease, while the p62 therapeutic use in the aged OXYS group reversed the AMD-like retinopathy and alleviated the pathological scenario. In particular, p62-treated rodents demonstrated a recovered RPE. The outcomes of p62 treatment also met the anti-inflammatory need to contrast with the AMD milieu since the plasmid was able to moderate macrophagal and microglial infiltration in the outer retina [53]. To recapitulate, p62 DNA relieved the AMD inflammatory setting, working against disease progression in young animals and aiding aged patients to ameliorate the overall clinical picture of the disease. The effect of the p62 plasmid resembles its effect on inflammation during osteoporosis and metabolic syndrome, as described above.

### 2.4. P62 Preventive and Therapeutic Effects against Tumors in Mice

As previously mentioned, the p62 multi-hub protein is involved in autophagic apparatus as an essential component to enhance diverse intracellular signaling routes comprising TRAF6, p38 and NF-κb signaling [28,54]. In addition, p62 was found to be overexpressed in several tumors. Based on these observations, earlier studies hypothesized that p62 was possibly induced by the Ras oncogene, and could be essential for the treatment of breast, prostate cancer and melanoma, facilitating tumor survival and expansion [55,56,57]. In line with this hypothesis, Venanzi et al. [57] showed that the p62 DNA intramuscularly administered in various models of allogenic murine tumor (B16 melanoma, Lewis lung carcinoma (LLC), S37 sarcoma and Ca755 breast carcinoma) exerted a notable anti-tumor effect both in prevention and in a therapeutic setting. Precisely, p62 DNA acts as a strong suppressor of all four mouse solid tumors mentioned above, but also an efficient anti-metastatic agent in LLC, S37 sarcoma and B16 melanoma mouse models [57].

Efforts are currently underway to clarify the mechanistic mode of action of p62 in mouse tumors. P62 upregulation observed in various tumors may be not the contributing case of tumor but, for instance, a mediator for its transformation and/or alteration and a potential sentinel for the recruitment of anti-inflammatory agents and immune cells.

### 2.5. P62-Encoding Plasmid as Therapeutic Agent in Dogs with Mammary Tumors

Canine mammary gland tumors (CMT) comprise a heterogeneous group of neoplasms, having the highest tumor incidence in intact female dogs, with an average diagnosis of malignancy at around 20–80% [58,59]. Indeed, in recent years, the balance points towards malignant instead of benign mammary tumors, a trend also detected in human breast tumors [60]. Surgical resection remains the indisputable main therapeutic approach for CMT and human breast cancer, but due to tumor recurrence and eventual metastases [58], the need for additional chemotherapy combined with targeted and non-targeted innovative treatments appears mandatory. Although most curative protocols efficient in human breast cancer have been also applied to dogs, not all of them found their way to being approval. For instance, adjuvants chemotherapy did not have the expected beneficial results in CMT [61,62] and immunotherapy still needs to be refined [63] due to the high CMT mortality: over 40% of patients [64]. Thus, in the last decade, the development of more accurate therapies remained a compelling task for field scientists. However, we should not overlook the fact that the safety and efficacy of novel drugs and vaccines in companion animals represent the missing piece prior to initiating human clinical trials [65].

In this context, the p62-encoding plasmid showed an efficient and safe therapy for mammary tumors in dogs. The trials involved dogs of different breads and with average age of 12 years. The response at the p62 DNA (doses of 0.75, 1.0 or 2.5 mg once a week for 3–10 weeks) was characterized by two phases: an increase in tumor size at the beginning of the administration (likely due immune cells infiltration) followed by tumor shrinkage. Interestingly, p62 DNA was able to cause a fibrotic encapsulation and reduction in tumors, both in mastectomy and inoperable groups. The authors claimed that a p62 DNA plasmid injection was efficient in feline mammary tumors [66].

Further studies conducted in dogs with mammary tumors, which addressed the evaluation of mechanisms that regulate the efficacy of p62 DNA against cancer, indicate that the administration of a plasmid provokes a rearrangement in the tumor microenvironment decreasing both the size and the aggressivity of the tumor. Intriguingly, p62 treatment restructured the tumor extracellular matrix through a significant increase in α-SMA and collagen type 3 deposition, rendering the tumor less aggressive and more easily treatable with chemotherapeutic agents [67].

A thought-provoking query arises concerning the ability of the p62 plasmid to stimulate the adaptive immune response. To meet this requirement, mice with severe combined immunodeficiency (SCID), lacking T and B cells and the WT counterpart (with an intact immune system), were challenged with B16 melanoma cells and treated with p62 plasmid. The results revealed that the adaptive immune system was necessary for the anti-cancer activity of the p62 [67]. Accordingly, p62 administration in mouse models with tumors provokes lymphocytes enrollment in the cancer microenvironment [57].

## 3. Can a Human Anticancer Experimental Drug, p62-Encoding Plasmid, Become a Broad-Spectrum Anticancer Therapy for Companion Animals?

Cancer research is readily motivated to resolve two crucial requirements for cancer therapy: enhancing anti-cancer immune responses and alleviating the surrounding inflammatory foci. To accomplish this convoluted task, innovative chemo-radiotherapy and immunotherapy protocols combined with anti-inflammatory agents come into the multifaceted equation to fight cancer.

Recently, a study involving a cohort of human patients with breast, ovary, lung, renal cancer and melanoma revealed that treatment with p62 DNA plasmid (coined as “Elenagen” by the authors), has managed to inverse the inflammatory tumor microenvironment within a good safety profile, and thus without any significant hematotoxic, hepatotoxic or nephrotoxic effect or other systemic side effects (brain, heart, lung, liver, kidney, spleen, thyroid and thymus morphology and functions were also investigated after “Elenagen” administration) [68]. Interestingly, patients with progressive metastatic solid tumors, who have had five intramuscular inoculations of the p62 plasmid, partially restored their sensibility to chemotherapeutic protocols with a concurrent reduction in tumor progress from 8 up to 38 weeks [68]. In addition, it could be intriguing to study the combined anti-tumor action of a p62 DNA plasmid with immunotherapeutics, anti-cancer vaccines and checkpoint inhibitors, such as anti-CTLA-4 and anti-PD-1 [69] in humans and animals. In this context, a case report of a patient affected by an aggressive triple-negative breast cancer indicated that p62 DNA plasmid treatment in combination with the standard chemotherapeutics, cyclophosphamide, methotrexate and fluorouracil, was able to partially block cancer progression and permit progression-free survival for at least 19 weeks [70]. Furthermore, accumulated evidence supports the concept that the plasmid p62-encoding addition in chemotherapy protocols can moderates chronic inflammation, modify the tumor setting, and intensify the number of tumor-infiltrating lymphocytes [71]. Moreover, the chemotherapeutic gemcitabine, combined with the p62-plasmid, appears to be a promising therapeutic platform for advanced platinum-resistant ovarian cancer [72].

**Figure 2:**
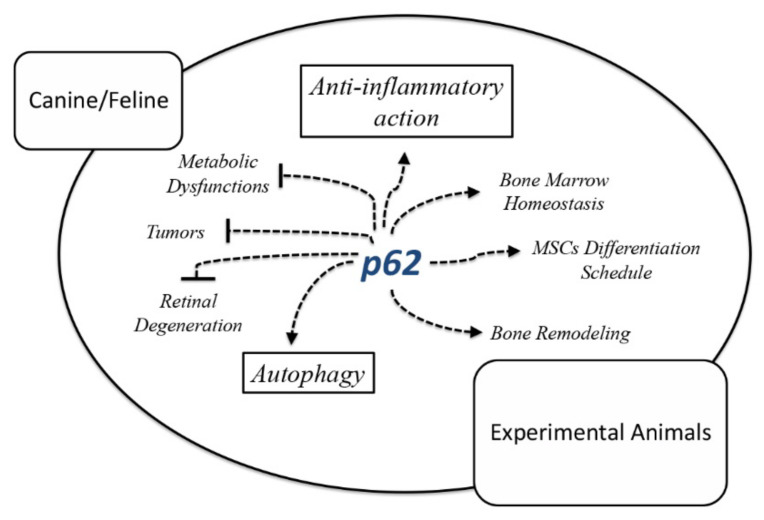
Multifarious action of p62 in experimental and domestic animals. P62 promotes MSCs differentiation toward osteoblasts, reduces adipocytes infiltration within bone marrow and controls bone remodeling processes in rodents. Furthermore, p62 DNA plasmid administration alleviates chronic inflammation and partially restores the pathological milieu in specific inflammation-based diseases.

Bearing in mind the p62-plasmid efficacy on breast cancer in dogs, it becomes logical to predict the p62-based curative potential within multi-therapy regimens in comparative oncology. Notably, only in the United States, the number of yearly canine cancer patients amounts to more than 4 million [73,74]. Research efforts revealed that dogs appear to develop bone and soft tissue sarcomas and hematopoietic cancers with a higher incidence than humans [73]. The study of various canine tumors, such as urothelial carcinoma; glioma, melanoma, lung and breast cancers; lymphoma; and osteosarcoma, which share similar molecular aspects with humans, have had beneficial impacts on animals and translational research [74]. Thus, spontaneous tumor studies on pet dogs have found their way into translational medicine, and a deeper understanding of their molecular profile has widely contributed to human tumor biology [74,75]. Due to the structural and functional relationship between canine and human cancers, the big query is whether specific human anti-cancer experimental drugs may have similar therapeutic outcomes in pet animals. For instance, diverse immune checkpoint inhibitors against osteosarcoma have been evaluated in human oncology and parallel research is ongoing on canine osteosarcoma [76,77]. Recently, a canine-specific monoclonal antibody derived from rituximab, an antibody used to treat human B cell lymphomas [78,79], as well as an anti-canine CD19-targeted monoclonal antibody against B cell pathologies, are also being considered [80]. Both antibodies are ushering in a new era of common immunotherapeutic strategies for human and pet malignancies. Drug combination protocols that include traditional chemotherapeutics, cyclooxygenase inhibitors and/or immune checkpoint blockages, are currently undergoing clinical trials for human and canine urothelial carcinoma. Lung carcinoma, glioma and melanoma are further examples of malignancies that share a common human/dog platform for medicines development [74]. In this context, the acclaimed therapeutic properties of the p62 DNA plasmid in humans may redirect the p62 research interest toward comparative oncology (Figure 2). P62-based mono-therapy, or therapy within an anti-cancer formulation, may provide a valuable anti-inflammatory and anti-metastatic platform in animal patients. Although there is a meaningful difference between p62/SQSTM1 gene sequences in dogs and humans, the p62 DNA plasmid action shares a similar therapeutic profile with the cancer microenvironments humans and pet dogs [81]. Overall, the p62 DNA plasmid could become a considerable therapeutic or preventive agent for inflammatory-based diseases of companion animals and could contribute to a combined anti-cancer treatment.

## 4. Conclusions

P62 is defined as the odd-jobber protein, able to orchestrate autophagy, coordinate stem cell differentiation, quench inflammation and actively participate in tumor stroma recovery through immune cells recruitment and anti-metastatic activity. P62 DNA plasmid intramuscular administration turned out to be a significant therapeutic agent against osteopathies, bone marrow dysfunctions, neurodegenerative ocular and metabolic diseases and mammary tumors in laboratory models. In some cases, the effectiveness of this plasmid was also tested in prevention protocols e.g., against osteopenia in mice with promising results. Research efforts in this area seems to be imperative. The data obtained portrayed p62 as a valuable player for clinical applications in veterinary practice. Inflammation- and inflamm-aging-related diseases in animals represent a poorly explored clinical dimension that deserves more research attention. The anti-inflammatory and anti-metastatic actions of the p62 plasmid may be a critical framework upon which novel anti-cancer protocols could be based. Many spontaneous pathological settings, e.g., cutaneous, ocular, and intestinal diseases, may also benefit from the therapeutic potential of the p62 plasmid.

P62 function as a therapeutic mediator and a versatile molecule to combine in experimental protocols against various inflammatory-based diseases in domestic animals will be greatly supported in the improvement of clinical trials and translational research.

## Data Availability

No new data were created or analyzed in this study. Data sharing is not applicable to this article.

## Animal Welfare Statement

No ethical approval was required as this is a review article with no original research data.
